# Supplementary material for: The RyR1 P3528S Substitution Alters Mouse Skeletal Muscle Contractile Properties and RyR1 Ion Channel Gating
Source: Int J Mol Sci. 2023 Dec 28;25(1):434. doi: 10.3390/ijms25010434 (PMC10778724; doi:10.3390/ijms25010434)
Supplement: Supplementary file 1 [file ijms-25-00434-s001.zip › ijms-2728058-supplementary.pdf]

Supplementary Data

The RyR1 P3528S Substitution Alters Mouse Skeletal Muscle Contractile Properties and RyR1 Ion Channel Gating

Chris G. Thekkedam [1,†], Travis L. Dutka [2], Chris Van der Poel [3], Gaetan Burgio [4] and Angela F. Dulhunty [1,*]

[1] Eccles Institute of Neuroscience, John Curtin School of Medical Research, Australian National University, Acton, ACT 2601, Australia; c.thekkedam@victorchang.edu.au

[2] Department of Animal, Plant and Soil Sciences, School of Agriculture, Biomedicine and Environment (SABE), La Trobe University, Melbourne, VIC 3086, Australia; t.dutka@latrobe.edu.au

[3] Department of Microbiology, Anatomy, Physiology and Pharmacology, School of Agriculture, Biomedicine and Environment, La Trobe University, Melbourne, VIC 3086, Australia; c.vanderpoel@latrobe.edu.au

[4] Division of Genome Sciences and Cancer, John Curtin School of Medical Research, Australian National University, Acton, ACT 2601, Australia; gaetan.burgio@anu.edu.au

* Correspondence: angela.dulhunty@anu.edu.au

† Present address: Victor Chang Institute, Lowy Packer Building, 405 Liverpool St, Darlinghurst, NSW 2010, Australia.

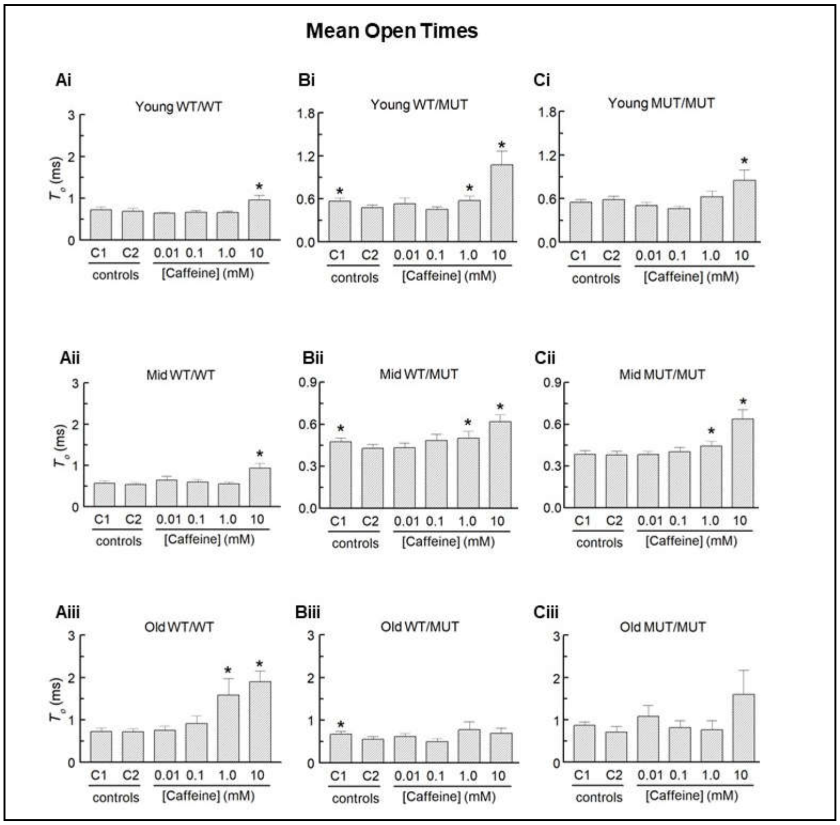

Figure S1. The influence of the RyR1 P3528S mutation on the changes in the mean open time of RyR1 channels in response to a reduction in cytoplasmic [Ca^{2+}] from 1 μM to 300 nM (C1 to C2) and to the addition of caffeine in increasing concentrations from 10 μM to 10 mM. A-C. Graphs of the average channel mean open time (T_o in ms) with 1 μM *cis* Ca^{2+} (C1), 300 nM *cis* Ca^{2+} (C2) and then after progressive increases in cytoplasmic [caffeine] to 10 μM, 100 μM, 1 mM and 10 mM. Average T_o is shown for channels from WT/WT mice (column **A**), WT/MUT mice (column **B**) and MUT/MUT mice (column **C**). Average T_o is shown for channels from young (row **Ai-Ci)**), middle (row **Aii-Cii**) and old (row **Aiii-Ciii**) aged mice. T_o values obtained at +40 mV and -40 mV are included in the average values, shown as mean ± sem. Young WT/WT, n=14; young WT/MUT n=16; young MUT/MUT n=12; middle aged WT/WT n=16; middle aged

WT/MUT n=16; middle aged MUT/MUT n=14; old WT/WT n=8; old WT/MUT n=8; old MUT/MUT n=6. *, significantly different from C2 (300 nM *cis* Ca^{2+}).

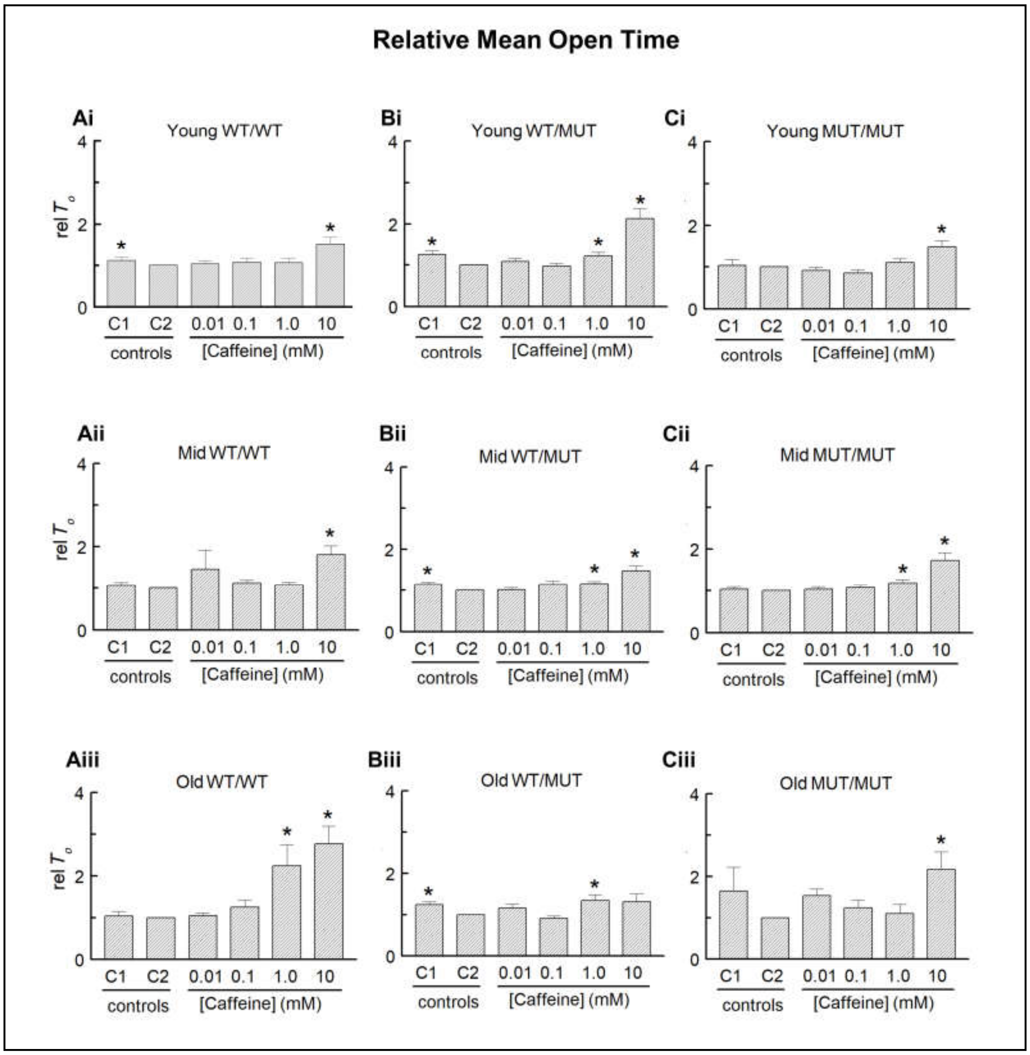

Figure S2. **The influence of the RyR1 P3528S mutation on the changes in the relative mean open time of RyR1 channels in response to a reduction in cytoplasmic [Ca^{2+}] from 1 μM to 300 nM (C1 to C2) and to the addition of caffeine in increasing concentrations from 10 μM to 10 mM.** **A-C**. Graphs of average relative mean open time (rel T_o) with 1 μM *cis* Ca^{2+} (C1), 300 nM *cis* Ca^{2+} (C2) and then after progressive increases in cytoplasmic [caffeine] to 10 μM, 100 μM, 1 mM and 10 mM. Average rel P_o is shown for channels from WT/WT mice (column **A**), WT/MUT mice (column **B**) and MUT/MUT mice (column **C**). Data is shown for channels from young (**Ai - Aiii**), middle (**Bi - Biii**) and old (row **Ci - Ciii**) aged mice. Rel T_o values obtained at +40 mV and -40 mV are included in the average values which are shown as mean ± sem. The numbers of observations in each group were as follows. Young WT/WT, n=14; young WT/MUT n=16; young MUT/MUT n=12; middle aged WT/WT n=16; middle aged WT/MUT n=16; middle aged MUT/MUT n=14; old WT/WT n=8; old WT/MUT n=8; old MUT/MUT n=6. *, significantly different from C2 (300 nM cis Ca^{2+}).

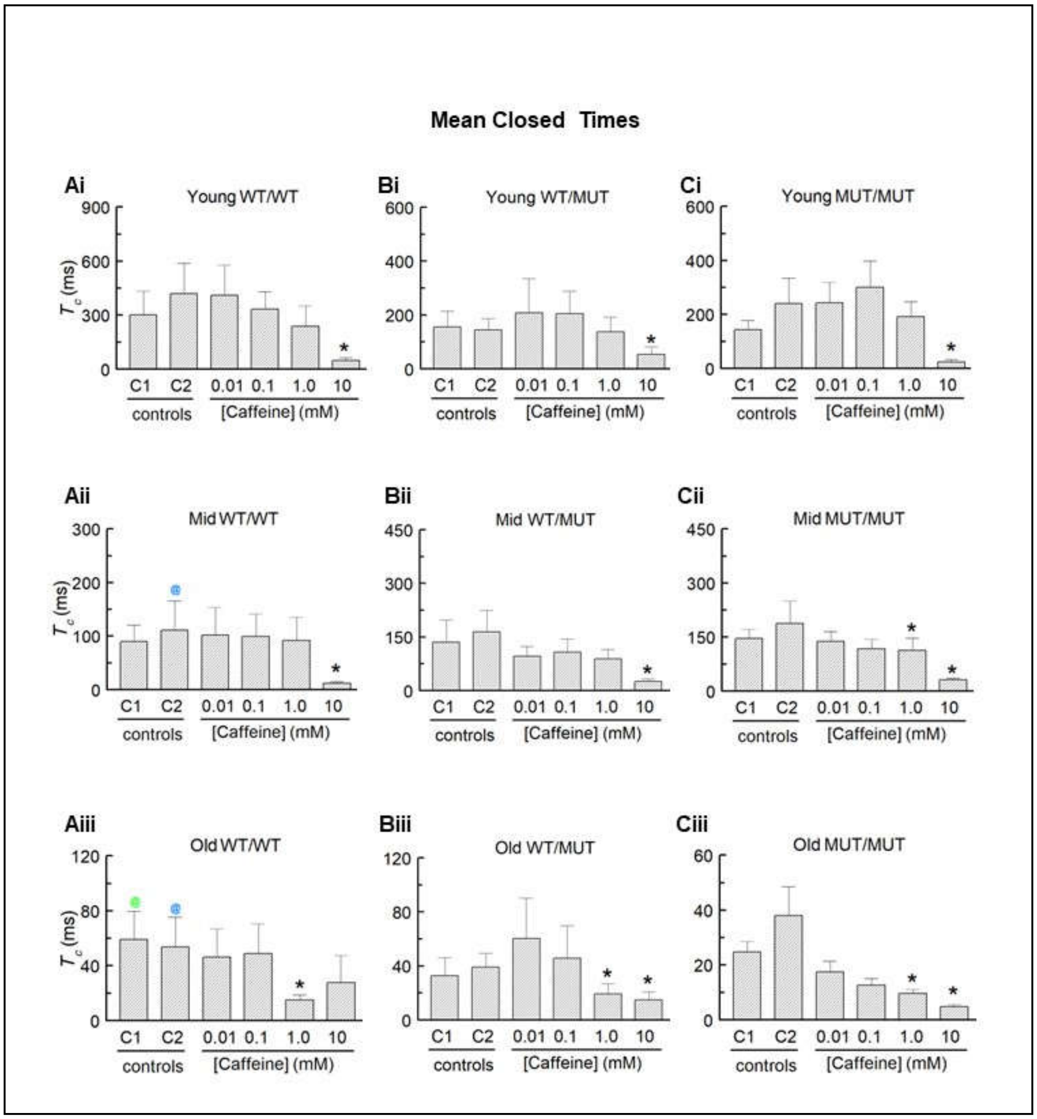

Figure S3. **The influence of the RyR1 P3528S mutation on the changes in the mean closed duration of RyR1 channels in response to a reduction in cytoplasmic [Ca^{2+}] from 1 µM to 300 nM (C1 to C2) and to the addition of caffeine in increasing concentrations from 10 µM to 10 mM.** **A-C**. Graphs of average mean closed time (T_c) with 1 µM *cis* Ca^{2+} (C1), 300 nM *cis* Ca^{2+} (C2) and then after progressive increases in cytoplasmic [caffeine] to 10 µM, 100 µM, 1 mM and 10 mM. Average T_c is shown for channels from WT/WT (column **A**), WT/MUT (column **B**) and MUT/MUT (column **C**) mice. Data is shown for channels from young (**Ai - Aiii**), middle (**Bi - Biii**) and old (row **Ci - Ciii**) aged mice. T_c values obtained at +40 mV and -40 mV are included in the average values which are shown as mean ± sem. The numbers of observations in each group were as follows. Young WT/WT, n=14; young WT/MUT n=16; young MUT/MUT n=12; middle aged WT/WT n=16; middle aged WT/MUT n=16; middle aged MUT/MUT n=14; old WT/WT n=8; old WT/MUT n=8; old MUT/MUT n=6. *, significantly different from C2 (300 nM *cis* Ca^{2+}); @, significantly different from C1 (1 µM cis Ca^{2+}) in channels from young WT/WT mice; @, significantly different from C2 (300 nM cis Ca^{2+}) in channels from young WT/WT mice.

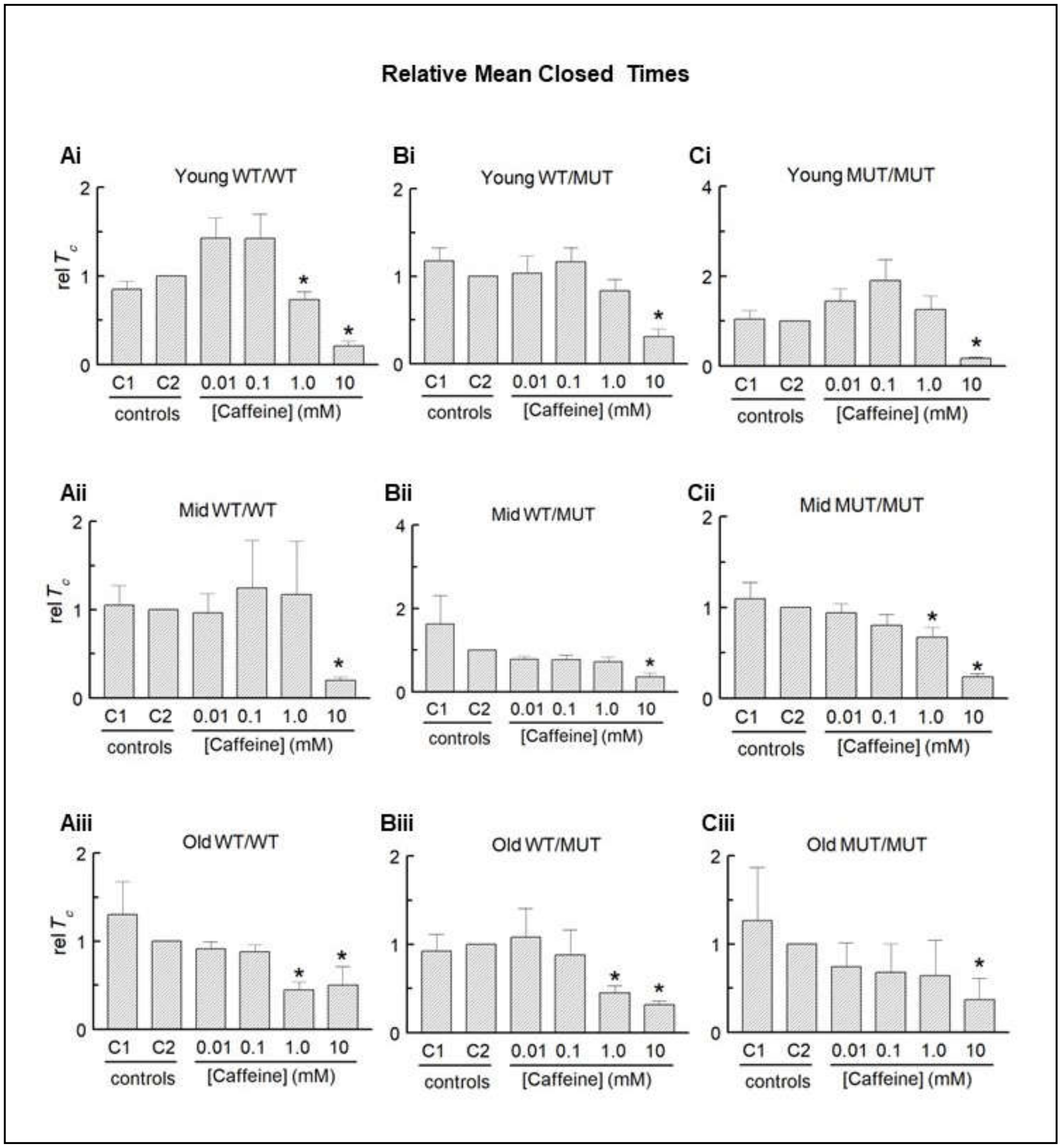

Figure S4. The influence of the RyR1 P3528S mutation on the changes in the relative mean closed duration of RyR1 channels in response to a reduction in cytoplasmic [Ca^{2+}] from 1 μM to 300 nM (C1 to C2) and to the addition of caffeine in increasing concentrations from 10 μM to 10 mM. A-C. Graphs of average relative mean closed time (rel T_c) with 1 μM *cis* Ca^{2+} (C1), 300 nM *cis* Ca^{2+} (C2) and then after progressive increases in cytoplasmic [caffeine] to 10 μM, 100 μM, 1 mM and 10 mM. Average rel T_c is shown for channels from WT/WT mice (column **A**), WT/MUT mice (column **B**) and MUT/MUT mice (column **C**). Data is shown for channels from young (**Ai - Aiii**), middle (**Bi - Biii**) and old (row **Ci - Ciii**) aged mice. Rel T_c values obtained at +40 mV and -40 mV are included in the average values which are shown as mean ± sem. The numbers of observations in each group were as follows. Young WT/WT, n=14; young WT/MUT n=16; young MUT/MUT n=12; middle aged WT/WT n=16; middle aged WT/MUT n=16; middle aged MUT/MUT n=14; old WT/WT n=8; old WT/MUT n=8; old MUT/MUT n=6. The asterisks indicate significant differences from the C2 control values obtained with 300 nM *cis* Ca^{2+}. *, significantly different from C2 (300 nM cis Ca^{2+}).

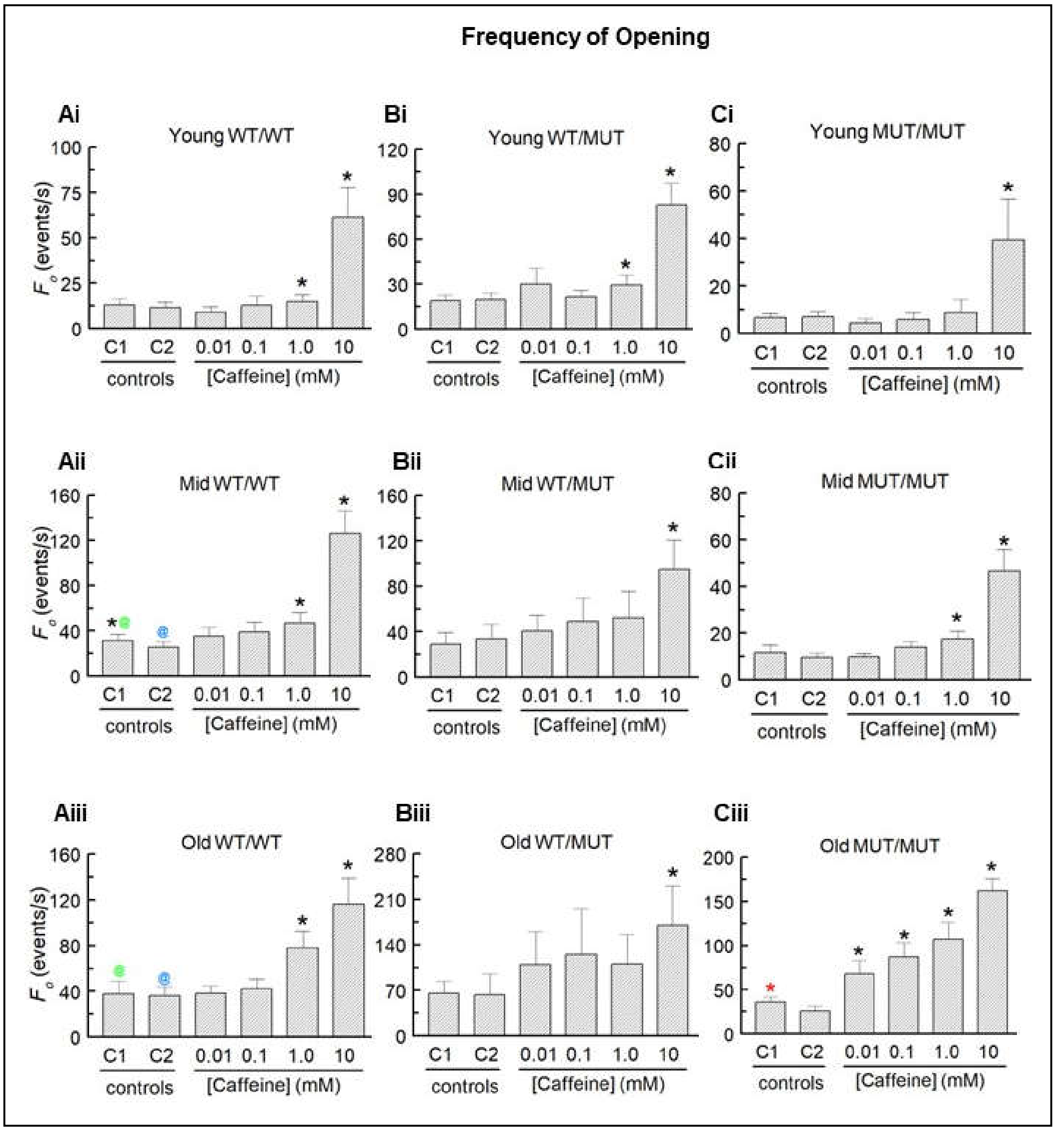

Figure S5. **The influence of the RyR1 P3528S mutation on the changes in the frequency of open events in RyR1 channels in response to a reduction in cytoplasmic [Ca^{2+}] from 1 µM to 300 nM (C1 to C2) and response to the addition of caffeine in increasing concentrations from 10 µM to 10 mM.** **A-C**. Graphs of average frequency (F_o) of channel open events with 1 µM *cis* Ca^{2+} (C1), 300 nM *cis* Ca^{2+} (C2) and then after progressive increases in cytoplasmic caffeine concentrations to 10 µM, 100 µM, 1 mM and 10 mM. Average F_o is shown for channels from WT/WT mice (column **A**), WT/MUT mice (column **B**) and MUT/MUT mice (column **C**). Data is shown for channels from young (**Ai - Aiii**), middle (**Bi - Biii**) and old (row **Ci - Ciii**) aged mice. F_o values obtained at +40 mV and -40 mV were included in the average values which are shown as mean ± sem. The numbers of observations in each group were as follows. Young WT/WT, n=14; young WT/MUT n=16; young MUT/MUT n=12; middle aged WT/WT n=16; middle aged WT/MUT n=16; middle aged MUT/MUT n=14; old WT/WT n=8; old WT/MUT n=8; old MUT/MUT n=6. * or *, significantly different from corresponding C2 value (300 nM cis Ca^{2+}); @, significantly different from C1 (1 µM cis Ca^{2+}) in channels from young WT/WT mice; @, significantly different from C2 (300 nM cis Ca^{2+}) in channels from young WT/WT mice.

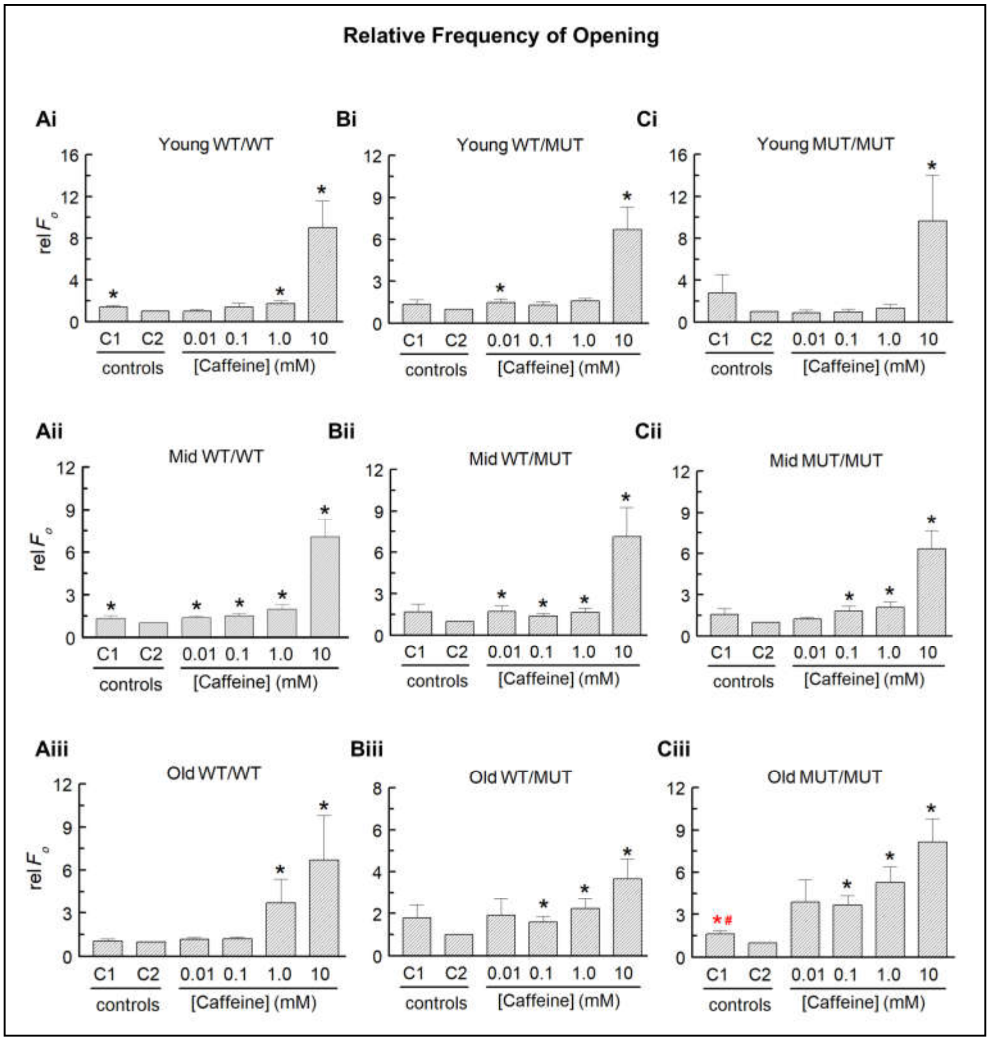

Figure S6. **The influence of the RyR1 P3528S mutation on changes in the relative frequency of open events in RyR1 channels in response to a reduction in cytoplasmic [Ca²⁺] from 1 µM to 300 nM (C1 to C2) and response to the addition of caffeine in increasing concentrations from 10 µM to 10 mM.** **A-C**. Graphs of average relative event frequency (rel F_o) with 1 µM *cis* Ca^{2+} (C1), 300 nM *cis* Ca^{2+} (C2) and then after progressive increases in cytoplasmic caffeine concentrations to 10 µM, 100 µM, 1 mM and 10 mM. Average rel F_o is shown for channels from WT/WT mice (column **A**), WT/MUT mice (column **B**) and MUT/MUT mice (column **C**). Data is shown for channels from young (**Ai - Aiii**), middle (**Bi - Biii**) and old (row **Ci - Ciii**) aged mice. Rel F_o values obtained at +40 mV and -40 mV were included in the average values which are shown as mean ± sem. The numbers of observations in each group were as follows. Young WT/WT, n=14; young WT/MUT n=16; young MUT/MUT n=12; middle aged WT/WT n=16; middle aged WT/MUT n=16; middle aged MUT/MUT n=14; old WT/WT n=8; old WT/MUT n=8; old MUT/MUT n=6. * or *, significantly different from C2 (300 nM cis Ca^{2+}); # significantly different from old WT/WT.
